# Short-term surgical outcomes in patients with unilateral complete cleft lip and palate after presurgical nasoalveolar molding therapy: A three-dimensional anthropometric study

**DOI:** 10.3389/fped.2022.1101184

**Published:** 2022-12-30

**Authors:** Jiayi Yin, Shiming Zhang, Ning Huang, Bing Shi, Qian Zheng, Chao Yang

**Affiliations:** ^1^ State Key Laboratory of Oral Diseases and National Clinical Research Center for Oral Diseases and Department of Oral and Maxillofacial Surgery, West China Hospital of Stomatology, Sichuan University, Chengdu, China; ^2^ State Key Laboratory of Oral Diseases and National Clinical Research Center for Oral Diseases and Department of Orthodontics, West China Hospital of Stomatology, Sichuan University, Chengdu, China

**Keywords:** nasoalveolar molding (NAM), cleft lip and palate, anthropometry, nasal deformity, short time observation

## Abstract

**Objective:**

This brief research report aimed to evaluate the short-term efficacy of presurgical nasoalveolar molding (PNAM) therapy on the nasolabial morphology three dimensionally in patients with non-syndromic complete unilateral cleft lip and palate (UCLP).

**Methods:**

Thirty-six patients with non-syndromic complete unilateral complete cleft lip and palate were enrolled retrospectively and categorized into 2 groups: 18 patients who had received PNAM treatment (PNAM group) and 18 age-matched patients who have not receive PNAM treatment (no PNAM group) from 2017 to 2021. The average starting age for PNAM therapy was 18.33 days, and the average PNAM treatment duration was 99.08 days. Twelve nasolabial parameters were measured to compare the postsurgical outcomes of two groups.

**Results:**

In PNAM groups, cleft width, vertical distance between double Crista philtri and columellar deviation were reduced compared to that in no PNAM group. And nostril height was larger than that in no PNAM group. The differences between two groups were statistically significant (*p* < .05). There were no statistical differences in columellar length, nostril width and bi-alar width between two groups. However, the nostril width on cleft side in PNAM group was decreased by an average of 1.1 mm.

**Conclusion:**

Our result indicated that PNAM therapy decreased cleft width and vertical distance between Crista philtri. It also increased nasal symmetry by decreasing columellar deviation, increasing nostril height.

## Introduction

The difficulties of primary surgical treatment for unilateral complete cleft lip (UCLP) are the discrepancies and displacement of the nasomaxillary morphology, such as wide cleft gap and the displacement of alveolar segments. Presurgical nasoalveolar molding (PNAM), established by Grayson et al. (1), is a non-surgical method of reducing cleft width, aligning the alveolar segments and deforming nasal lower lateral cartilages to minimize the severity of cleft deformity before primary unilateral cleft lip repair and palatoplasty and consequently improving surgical outcomes. In the past few decades, there have been many reports documented favorable efficacy of PNAM in decreasing cleft avelo (2–5).

Precise imaging of craniofacial malformation is a crucial precondition for UCLP patients' treatment. The introduction of three-dimensional (3D) measurements such as stereophotogrammetry (6–9) and 3D laser scanning (10–13), facilitates the recording and assessment of CLP patients’ dento-maxillofacial morphology compared to traditional direct anthropometric and two-dimensional measurement based on photographs. However, the 3D reconstruction of dento-maxillofacial profile might have distortion due to insufficient instrument precision, change in patient's facial expression and head movement etc. The profile deviation, stringent specification of the measuring device and high expense limit the promotion in clinical application. Another frequently used method is facial plaster casts. It is economic, accessible and guarantees the verisimilitude and accuracy of facial details. Therefore, the present study used dental plaster casts to quantify facial landmarks of infants with complete UCLP and retrospectively evaluate the short-term effect of PNAM therapy on nasolabial symmetry.

## Materials and methods

### Patients

Patient data were retrieved from the electronic records of the department of cleft lip and palate at West China Hospital of Stomatology, Sichuan University. 18 complete UCLP patients (11 boys, 7 girls) aged 3 to 6 months who received PNAM from November 2017 to November 2021 were extracted for the study. Besides, 18 age-matched complete UCLP patients (10 boys, 8 girls) who didn't undergo any non-surgical treatment were enrolled as control group. The participants were selected for inclusion in the present study based on the following criteria: (1) infants with non-syndromic complete UCLP; (2) no other co-existing craniofacial malformations; (3) first consultation during the neonatal period, (4) written consent of one or both parents for any clinical-surgical practice.

**Figure 1:**
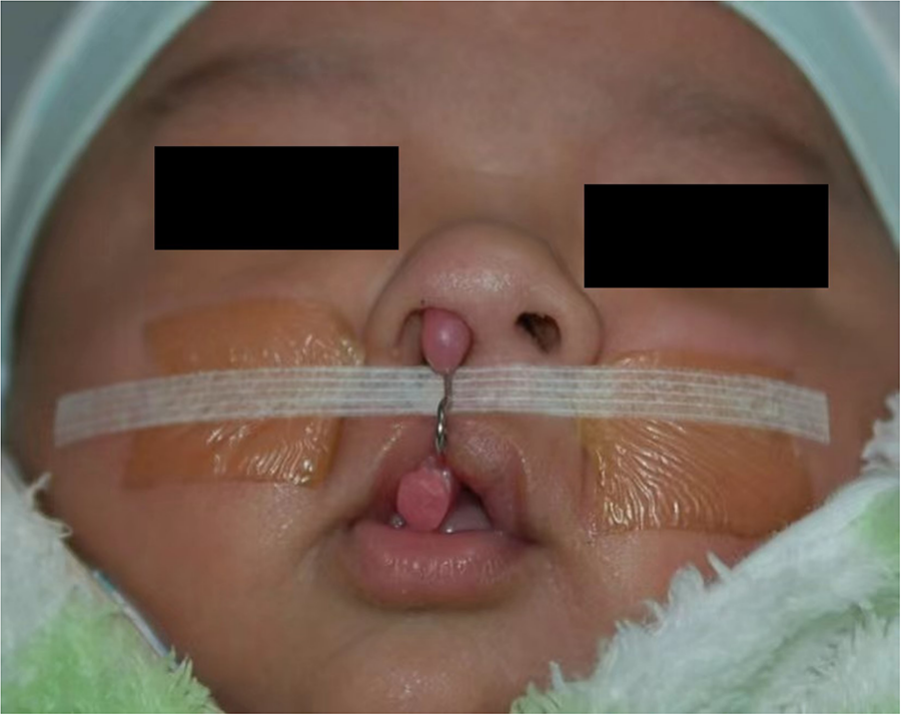
A study patient wearing the presurgical nasoalveolar molding (PNAM) appliance.

The average age of the patients upon commencing PNAM therapy was 18.33 days (range 2 to 32 days), and the average therapy duration was 99.08 days (range 65 to 126 days). The procedure of PNAM therapy was basically in accordance with the Grayson's technique (1). There were two main differences: first, nasal stent was added to the dental plate when the patients adapted to it (The infant only spent 7–14 days to completely adapt to the nasal stent) and then the nasal stent was adjusted weekly; second, the PNAM appliance was fixed in position with denture adhesive. A horizontal tape (3M Steri strips-1/4 Inch) was placed at the base of nose, stretching the lip segments toward each other. Artificial skin was applied to each cheek to avoid skin irritation created by adhesive tape (Figure 1).

All impressions were taken from participated infants under general anesthesia and the casts were fabricated with dental plaster. The bases of the facial casts were trimmed and adjusted to make the planes of all casts identical. All casts were marked with computerized random numbers for the subsequent blinded measurement. All PNAM therapy and casts were accomplished by the same author (C.Y). The protocol for the current study was reviewed and approved by the Institutional Review Board of West China Hospital of Stomatology, Sichuan University (No. WCHSIRB-D-2016–084R1).

### Landmarks and measurement

The facial anatomical landmarks were identified on each cast (Table 1). The horizontal reference line was constructed by connecting the endocanthion points (Enr-Enl). Clefts were standardized to the right side by “mirroring” the facial measurements of patients with left-sided clefts.

**Table 1:** The facial landmarks and descriptions.

Landmark	Description
Endocanthion right and left (Enr and Enl)	The inner commissure of palpebral fissures on right and left side
Alare right and left (Alr and All)	The lateral insertion of the nasal rim near the upper lip.
Subalare right and left (Sar and Sal)	The medial insertion of the nasal rim near the upper lip; the most caudal end of the nasolabial crease
Columella (C)	Most superior point on the midline of columella
Subnasale (Sn)	The Midpoint of columella base where borders of nasal septum and upper lip meet
Crista philtri right and left (Cphr and Cphl)	The top points of Cupid's bow on right and left side
Cheilion right and left (Chr and Chl)	The most lateral labial commissure point on the right and left
Labrale superius (Ls)	The midpoint of Cupid's bow

**Figure 2:**
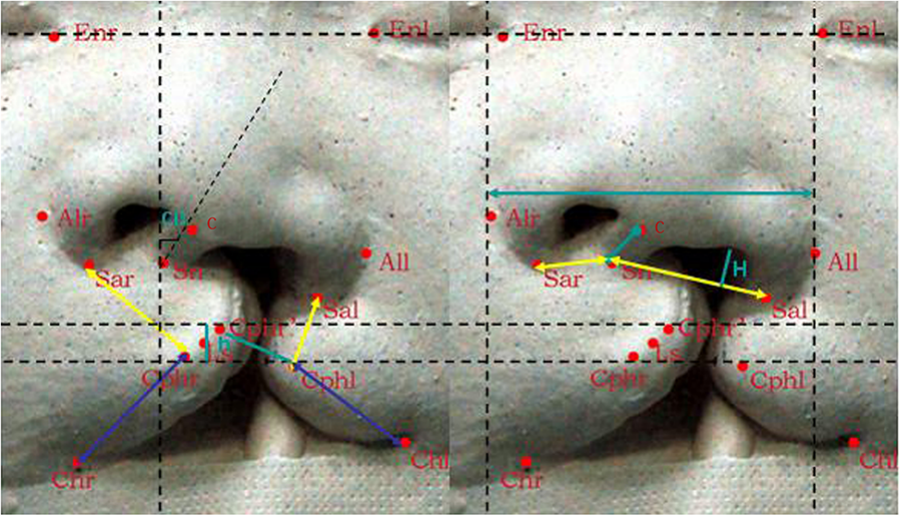
The horizontal reference line and facial anthropometric parameters. The horizontal reference line(Enr-Enl), non-cleft/cleft lip height (Sal-Cphl;Sar-Cphr),non-cleft/cleft lip length (Cphl-Chl;Cphr-Chr), cleft width (Cphr-Cphr’), vertical distance between double Crista philtri (h), columellar deviation (CD), columellar length (Sn-C), non-cleft/cleft nostril width (Sn-sar;Sn-sal), cleft nostril height(H); bi-alar width(All-Alr).

Twelve parameters were assessed to evaluate nasolabial symmetry: non-cleft/cleft lip height (Sal-Cphl; Sar-Cphr), non-cleft/cleft lip length (Cphl-Chl; Cphr-Chr), cleft width (Cphr-Cphr'), vertical distance between Crista philtri left and right (h), columellar deviation (CD), columellar length (Sn-C), non-cleft/cleft nostril width (Sn-sar;Sn-sal), cleft nostril height(H); bi-alar width(all-alr) (Figure 2).

The casts were measured directly using the electronic vernier caliper and protractor. The measurements were completed and repeated the three times by a single operator (S.L) on three different occasions.

Numerical data are presented as means ± SDs. Paired t-test was performed to compare the outcomes of PNAM group and no PNAM group. The statistical analyse was performed using IBM SPSS Statistics 20.0 (IBM Corp., Armonk, NY, USA). The *p* value <0.05 was considered as statistical significance.

## Results

All results are shown in Table 2. The cleft width reduced significantly (*p *< 0.001), resulting in better approximated lips. The vertical distance between Crista philtri demonstrated significant decrease in PNAM group (*p *< 0.001). However, lip height and lip length on both sides were not significantly different. In the assessment of nasal morphology, the PNAM group showed significant improvement in columellar deviation (*p *< 0.001). Columellar length and nostril width on cleft and non-cleft side did not show significant difference between two groups (*p *= 0.167, *p *= 0.847 and *p *= 0.628 respectively). The mean value of columellar length of PNAM group was higher compared to no PNAM group. The mean value of nostril width on non-cleft side was almost identical between the two groups while the mean value of nostril width on cleft side in the PNAM group was decreased by an average of 1.1 mm compared to no PNAM group. The cleft nostril height was larger in PNAM group, and the difference between two groups was statistically significant (*p *< 0.001). The mean value of bi-alar width exhibited non-statistically significance between two groups.

**Table 2:** Comparison of the nasal symmetry between PNMD group and np PNMD group.

Measurements	Mean ± Standard Deviation (mm)		*p* value
	No PNAM group	PNAM group	
Sal-cphl	8.52 ± 0.96	8.26 ± 2.02	0.694
Sar-cphr	10.92 ± 1.18	10.98 ± 1.02	0.775
Chl-Cphl	13.12 ± 1.44	13.28 ± 1.83.	0.847
Chr-Cphr	16.01 ± 2.14	18.83 ± 2.31	0.628
Cphl-Chpr′	15.73 ± 2.97	11.14 ± 1.53	0.038^a^
h	3.68 ± 1.26	2.58 ± 0.42	<0.001^a^
CD	49.30 ± 10.0	30.73 ± 7.24	<0.001^a^
Sn-C	4.31 ± 0.62	3.92 ± 0.68	0.167
Sn-Sar	10.68 ± 0.37	10.34 ± 1.24	0.715
Sn-Sal	20.35 ± 2.68	18.46 ± 2.69	0.088
H	3.02 ± 1.10	4.80 ± 0.83	<0.001^a^
Al1-Alr	33.38 ± 2.94	32.40 ± 2.59	0.418

All results based on paired sample *t* tests.

^a^ Measurements with ^a^ indicate a statistical significance (*p* < 0.05).

## Discussion

The prime aim of presurgical treatment in patients with UCLP is rehabilitating the deformed dento-maxillofacial components to allow for better surgical outcomes and reduced need for secondary revisions (14, 15). The present study was conducted to assess the efficacy of PNAM on short-term nasolabial symmetry before primary UCLP repair and the results demonstrated improvement in cleft width, vertical distance between double Crista philtri, columellar deviation and nostril height on cleft side in PNAM-treated patients. The lip taping during PNAM therapy approximated the lip reduction cleft width segments, improved lip symmetry and centralized the columella. The nasal stent contributed to the significant increase of nostril height on cleft side by reshaping the collapse alar cartilage. The columellar length, nostril width on both sides and bi-alar width showed no statistical difference in the present study. Though the current study demonstrated that PNAM group had improved nasolabial outcomes after the procedure in comparison with no PNAM group and the short-term effectiveness has been corroborated by numerous studies (16, 17), the post-surgical outcomes and long-term sustenance of facial symmetry have yet to be determined (8, 18, 19).

In previous studies, the most commonly used method for facial morphological analyses is two-dimensional (2D) photography (2, 18, 20, 21). The 2D photography offers the benefits of non-invasive, simple and quick facial morphology capturing. However, the photographs provide limited information about the 3D anatomical structure (22). The technique of plaster cast measurements in the current study is an easily reproducible and safe method for recording and evaluation of CLP patients' 3D facial morphology. This method also has some drawbacks, including errors during manual measurement and needs for storage space. Other 3D measurements such as stereophotogrammetry and laser scanning have been reported to be employed in PNAM therapy to acquire digitalized patients’ dento-maxillofacial structures and customize templates (23–25). Nevertheless, the equipment was not available for many CLP centers. To further validate the efficacy of PNAM, studies with large sample size and long-term follow-up with the application of 3D analysis were highly recommended.

## Data Availability

The raw data supporting the conclusions of this article will be made available by the authors, without undue reservation.
